# Circulation patterns and molecular epidemiology of human respiratory syncytial virus over five consecutive seasons in Morocco

**DOI:** 10.1111/irv.13203

**Published:** 2023-10-17

**Authors:** Abderrahman Bimouhen, Zakia Regragui, Fatima El Falaki, Hassan Ihazmade, Samira Benkerroum, Amal Barakat, Ahmed Rguig, Touria Benamar, Soumia Triki, Youssef Bakri, Hicham Oumzil

**Affiliations:** ^1^ Laboratory of Human Pathologies Biology, Faculty of Sciences Mohammed V University in Rabat Rabat Morocco; ^2^ National Influenza Center, Virology Department National Institute of Hygiene, Ministry of Health Rabat Morocco; ^3^ World Health Organization Regional Office for the Eastern Mediterranean Cairo Egypt; ^4^ Directorate of Epidemiology and Disease Control Ministry of Health Rabat Morocco; ^5^ World Health Organization Country Office of Morocco Rabat Morocco; ^6^ Center of human pathologies genomic, faculty of Medicine and Pharmacy Mohammed V university in Rabat Rabat Morocco; ^7^ Pedagogy and Research Unit of Microbiology, School of Medicine and Pharmacy Mohammed V University in Rabat Rabat Morocco

**Keywords:** circulation patterns, genotype, Morocco, phylogenetic analysis, respiratory syncytial virus

## Abstract

**Background:**

Respiratory syncytial virus (HRSV) is the leading cause of respiratory tract infections in infants and young children. we investigated the prevalence and characteristics of HRSV in Morocco and explored trends in circulating genotypes through partial G gene analysis of HRSV strains prevalent from 2012 to 2017.

**Methods:**

Respiratory samples were gathered from both outpatients and inpatients meeting ILI or SARI case definitions. The patients' ages varied from 1 month to 99 years old. Nucleic acids were extracted and HRSV type/subtype was detected by RT‐qPCR. A subset of positive samples was randomly selected in each epidemic year, the complete viral genome was sequenced, phylogenetic analysis was performed using the MEGA7 program and the genotypes were confirmed.

**Results:**

The 3679 specimens were collected from 2012 to 2017, of which 726 (19.7%) were positive for HRSV. The 35% (257/726) of HRSV‐positives were of the HRSV‐A subtype, while the HRSV‐B subtype accounted for 61% (442/726). The co‐infection rate was 3.7% (27/726). The virus circulates in a periodic pattern, where epidemics occur during the fall months through early spring. HRSV genotype was confirmed in 127 specimens (56 HRSV‐A and 71 HRSV‐B). Based on phylogenetic analysis, all HRSV‐A were ON1 genotype, and HRSV‐B were mostly BA9 genotype. HRSV‐B belonging to the BA10 genotype was detected in 2012 exclusively.

**Conclusions:**

BA9, BA10, and ON1 were the only HRSV genotypes detected between 2012 and 2017. Variations in the G gene amino acid chain were identified in local strains, which suggests an increased need for continuous genomic surveillance.

## 1 INTRODUCTION

A significant part of acute lower respiratory infections (ALRIs) in infants and young children worldwide are attributed to the respiratory syncytial virus (HRSV). These infections can be severe, resulting in pediatric hospitalizations and childhood deaths, mostly in developing countries.^1^ HRSV is also recognized as a major viral pathogen in the elderly, especially among those with chronic diseases and immunocompromised patients.^2^

HRSV is a non‐segmented, negative, single‐strand enveloped RNA virus, a member of the *Pneumovirinae* subfamily of the *Paramyxoviridae* family. The viral genome is about 15.2 kb in length, and it contains 10 genes that encode 11 proteins: nonstructural proteins NS1 and NS2; three nucleocapsid proteins N, P, and L; two matrix proteins, M and M2‐1; a transcription regulatory protein M2‐2; three transmembrane glycoproteins G, F, and small hydrophobic protein SH.^3^ HRSV is divided into two antigenic subtypes (A and B) that are mostly distinguished by differences in the viral attachment (G) protein and each has been divided into multiple genotypes.^4^ The sequence of the G gene is the most variable; thus, all molecular epidemiology studies of HRSV have focused on the second hypervariable region in the C‐terminal (HVR2) of the G protein and it has been used for virus genotyping.^5^ Fifteen genotypes of HRSV‐A (GA1 to −7, SAA1, CB‐A, NA1 to −4, ON1 and ON2) and 29 genotypes of HRSV‐B (BA1 to −14, GB1 to −5, SAB1 to −4, URU1, URU2, CB1, BA‐C, CB‐B, and THB) have been described.^6^, ^7^, ^8^, ^9^, ^10^ Among these genotypes, HRSV‐A ON1 and HRSV‐B BA with respectively 72 and 60 nucleotide duplications within the second hypervariable region of the G gene spread rapidly throughout the world and have been detected globally.^9^

The World Health Organization (WHO) launched a project for global respiratory syncytial virus surveillance in 2017 using the already long‐established, well‐functioning Global Influenza Surveillance and Response System (GISRS).^11^ The main expected key outcomes of this project were understanding the epidemiology and global circulation of HRSV strains, developing genetic resources of HRSV viruses in publicly accessible databases, determining highly risk groups for severe respiratory syncytial disease and building evidence that would enable countries to make informed policy decisions for introducing effective HRSV vaccines and monoclonal antibodies especially for infants and young children.

In Morocco, a sentinel‐based influenza surveillance system has been established since 1996^12^; however, data regarding other respiratory viruses remain scarce. Recent studies have attempted to describe circulation characteristics of respiratory viruses prevailing in Moroccan patients with Severe acute respiratory infections (SARI) or Influenza Like Illness (ILI), including the prevalence of HRSV.^13^ However, data on molecular epidemiology and phylogeny of HRSV strains in the country is still limited. Therefore, this study aims to investigate the prevalence of HRSV in patients with SARI or ILI, explore the seasonality of the virus, describe the clinical characteristics of patients with the infection, and to conduct phylogenetic and molecular analysis of different genotypes of HRSV strains in Morocco compared to what is observed in other countries in the world over the period 2012 to 2017.

## 2 MATERIALS AND METHODS

### 2.1 Sample and data collection

In this study, we retrospectively investigate respiratory samples collected from the existing influenza surveillance system for HRSV over five seasons from September 2012 to August 2017. As described earlier,^13^ virological surveillance for SARI was carried out throughout the year at nine public hospitals located in different regions of the country. Patients of all ages who met the World Health Organization's (WHO) criteria for SARI were enrolled,^14^ and medical staff recorded relevant epidemiological information (age, gender, medical history, clinical symptoms, and underlying conditions). The ILI virological surveillance system collects specimens and records data daily from the first five cases that meet the WHO's case definition for ILI^14^ in eight out‐patient public clinics and 50 private physicians located in nine major cities across the national territory. A total of 3679 Specimens were collected from patients, were stored at 4°C at the health facilities then sent to the National Influenza Center (NIC) within 48 h for immediate processing.

### 2.2 RNA extraction and diagnosis by real time RT‐qPCR

An initial volume of 400 μL of samples was extracted automatically using a High Pure Viral Nucleic Acid Kit and iPrep™ instrument, according to the manufacturer's instructions (Lifetechnologies, Carlsbad, USA). Ribonuclease P (RNase P) was considered as the internal control during specimen extraction. All samples were initially tested for Influenza A and B using a TaqMan® one‐step real‐time Reverse‐Transcriptase Polymerase Chain Reaction (RT‐qPCR) assay with specific primers and probes for influenza (CDC, USA), according to the manufacturer's recommendations. Detection of HRSV was performed in 5‐μL volume using Invitrogen® Superscript III Platinum® One‐step Reverse transcription polymerase chain reaction (RT‐qPCR), amplification and reaction conditions were made with an ABI 7500 Fast Sequence Detection System® in accordance with the non‐published protocols developed by Centers for Disease Control and Prevention (CDC; Atlanta, GA). The RT‐qPCR for Subtyping HRSV was performed with primers specific to the N gene of HRSV‐A and HRSV‐B; the amplification protocol was 50°C for 30 min, followed by 95°C for 2 min, and then by 45 cycles of 15 s at 94°C, and 35 s at 55°C.^15^ The reaction volume was 25 μL, including 5 μL of extracted RNA and 20 μL of reagent mix. Negative controls (Nuclease‐Free Water) were used in each reaction, and detection of a human gene (RNase P) was used as the internal control for the reaction.

### 2.3 Whole virus genome sequencing

As the country joined the second phase of a pilot study of WHO strategy for global HRSV surveillance project based on existing Influenza surveillance platform. Moroccan NIC performed detection and subtyping of HRSV over the year and periodic shipment of samples selected to provide temporal and geographical representation of HRSV circulating strains was sent to The Centers for Disease Control and Prevention (CDC, Atlanta), for whole genome sequencing (WGS) using the Illumina Nextera XTDNA library prep kit® on a MiSeq® platform (Illumina, San Diego, CA, USA) according to their established protocols. Fifty‐seven HRSV subtype‐A and 78 HRSV subtype‐B were randomly selected for sequencing.

Sequences were identified according to the newly adopted nomenclature of HRSV strains (below the Species Level) by the scientific community,^16^ as follows: HRSV/subgroup identifier/country code (ISO 3166‐1 alpha‐3)/unique sequence identifier/year of sampling.

### 2.4 Phylogenetic analysis

Among all relevant HRSV genes, the attachment glycoprotein gene holds the highest number of variations that can affect viral infection and transmission.^17^ Currently, genotyping of HRSV strains is based on the second hypervariable region of gene G (HVR‐2).^5^, ^10^ The phylogenetic analysis based on the alignment of HRSV G sequences, included sequences of HRSV‐A and HRSV‐B strains circulating in Morocco along with representative strains of known genotypes retrieved from the Genbank^18^ as at February 16, 2023. Identical sequences were detected and removed using “ElimDupes Duplicate Sequence Removal tool.”^19^ Multiple sequence alignments of the HVR‐2 of gene G compared to available reference genotypes were performed using the ClustalW algorithm. Phylogenetic trees were generated using the maximum likelihood algorithm in MEGA 7 software, and the bootstrapping for 1000 iterations was calculated to evaluate confidence estimates, only bootstrap values >60% were shown on the trees. The average pairwise distance (p‐distance) was calculated by the Tamura‐Nei model, gamma distributed, including transitions and transversions in the same software.

Comparison of the amino acid changes in the G protein of Moroccan HRSV‐A group to the prototype strain of ON1 genotype from Canada [JN257693] and the G protein of the HRSV‐B group to the prototype strain of BA genotype from Argentina [AY333364] was carried using BioEdit (version 7.1.3.0) sequence alignment editor (Ibis Biosciences, Carlsbad, CA).

### 2.5 Amino acid glycosylation site analysis

Potential N‐glycosylation sites (N‐X‐T/S, where X is not a proline) were predicted using NetNGlyc‐1.0 server^20^ and accepted if the glycosylation potential was ≥0.5. Where the O‐glycosylation residues were predicted using the NetOGlyc‐4.0 Server^21^ and accepted if the G‐score was ≥0.5.

### 2.6 Statistical analysis

To describe the temporal distribution of positive cases, we aggregated results as obtained by (RT‐qPCR) by calendar month and week. All enrolled patients' demographic, clinical, and virological data were entered into a database created using Epi Info™ 7.1 (CDC; Atlanta, USA) and summarized. Group comparisons were performed using *χ* ^2^ for the dependency/relationship between variables. *P*‐values of less than 0.01 were considered statistically significant. Data analysis was conducted using SPSS 20.0 (SPSS, Inc., Chicago, IL).

## 3 RESULTS

Between September 2012 and August 2017, we enrolled 3679 patients meeting the ILI or SARI case definition for five successive seasons. We collected 422 (11%), 101 (3%), 441 (12%), 1126 (31%), and 1589 (43%) samples during 2012/13, 2013/14, 2014/15, 2015/16 and 2016/17 influenza seasons (October–April) respectively. The proportion of samples collected from outpatients was 55.8% (2054/3679), slightly higher than those recruited from inpatients with 1625 samples (44.2%).

### 3.1 Demographic and clinical characteristics

A total of 1811 samples (49.2%) were collected from males and 1868 samples (50.8%) from females, with a male‐to‐female ratio of 0.97. Patients' ages ranged from 1 month to 99 years, with a mean age of 21 years, 11 months, and a median age of 11 years. The 1490 cases (40%) were younger than 5 years, 1391 cases (39%) were aged between 5–49 years, and 663 patients (18%) were older than 50 years. Among all sentinel sites included in this study, the highest number of patient specimens were collected from the Fes‐Meknes region (1121; 30%), followed by Rabat–Sale–Kenitra (876; 24%). Although samples were collected throughout the year, the majority of enrollments (2269; 62%) were recorded during the first quarter (January–March) of each season compared to other quarters (Table 1).

**TABLE 1:** Demographic and clinical characteristics of cases with influenza‐like illness (ILI) and severe acute respiratory infections (SARI) by respiratory syncytial virus infection status, Morocco, 2012–2017.

Demographic and clinical characteristic	HRSV‐positive *N* = 726 (19.7%) *n* (%)	HRSV‐negative *N* = 2953 (80.3%) *n* (%)	*P*‐value
***Age group (n = 3544)*** ^a^			
0–6 month	320 (44)	293 (10)	<0.001*
7–23 month	143 (20)	357 (12)	
2–4 year	86 (12)	291 (10)	
5–14 year	30 (4)	362 (12)	
15–49 year	71 (9)	928 (31)	
50–64 year	37 (5)	406 (14)	
>65 year	21 (3)	199 (7)	
unknown	18 (3)	117 (4)	
***Sex***			
Male	384 (53)	1427 (48)	0.027
Female	342 (47)	1526 (52)	
***Clinical feature***			
***Cough***			
Yes	652 (90)	2430 (82)	<0.001*
No	74 (10)	523 (18)	
***Fever***			
Yes	473 (65)	1936 (65)	0.434
No	253 (35)	1017 (35)	
***Sore throat***			
Yes	103 (14)	501 (16)	0.038
No	623 (86)	2452 (84)	
***Season***			
October–December	155 (21)	823 (28)	<0.001*
January–March	560 (77)	1709 (58)	
April–June	11 (2)	362 (12)	
July–September	0 (0)	59 (2)	
***Sentinel sites***			
Fes‐Meknes	240 (33)	881 (30)	<0.001*
Rabat‐Sale‐Kenitra	111 (15)	765 (26)	
Souss‐Massa (Agadir)	142 (20)	247 (8)	
Marrakech	52 (7)	269 (9)	
Tanger‐Tetouan	37 (5)	262 (9)	
Beni Mellal	79 (11)	219 (7)	
Oujda	37 (5)	161 (6)	
Casablanca	28 (4)	129 (4)	
Laayoune	0 (0)	14 (0)	

^a^ Some data variables from age have not been included in the analysis owing to incomplete data.

* Statistically significant at *P* < 0.01.

Cough was the most reported clinical symptom (84%, 3082/3679), followed by fever (65%, 2409/3679). A comparison of HRSV positive and negative cases alongside main clinical and demographic characteristics, that is, cough, fever, sore throat, sex, and age group, showed that the distribution of patients for cough was statistically significant (*P* < 0.001). However, we did not find a statistically significant difference in HRSV‐positive infections regarding fever (*P* = 0.434), sore throat (*P* = 0.038), or sex distribution (*P* = 0.027). Nevertheless, we found that the age group of patients and hospitalization status were statistically significantly related using the chi‐square test with a *P* < 0.001 (Tables 1 and 2).

**TABLE 2:** Influenza and HRSV detection rate among ILI and SARI patients, Morocco, 2012–2017.

Virus detection by qRT‐PCR N(%)	Syndrome			*P*‐value
	Total *N* = 3679	SARI *N* = 1625	ILI *N* = 2054	
HRSV‐Negative	2953 (80)	1127 (69)	1826 (89)	<0.001*
HRSV‐Positive	726 (20)	498 (31)	228 (11)	
Influenza virus‐Negative	2867 (78)	1458 (90)	1409 (69)	<0.001*
Influenza virus‐Positive	812 (22)	167 (10)	645 (31)	

* Statistically significant at *P* < 0.01.

### 3.2 Detection of HRSV and flu, the proportion of positives

All enrolled patients' respiratory samples were analyzed by RT‐qPCR for influenza viruses A/B and HRSV. Of the 3679 specimens tested from both ILI and SARI cases, a total of 726 specimens were positive for HRSV by RT‐qPCR, resulting in a detection rate of 19.7% (726/3679). Influenza frequency was 22.1% (812/3679), of which 34.1% (277/812) were Influenza A H1N1 pdm09, 33.0% (268/812) were Influenza A H3N2, and 32.8% (267/812) were Influenza B.

Of 2054 ILI cases, 645 (31%) tested positive for influenza A/B, and 228 (11%) were positive for HRSV. While Of 1625 SARI cases, 498(31%) were positive for HRSV and 167(10%) for influenza virus (Table 2).

**FIGURE 1:**
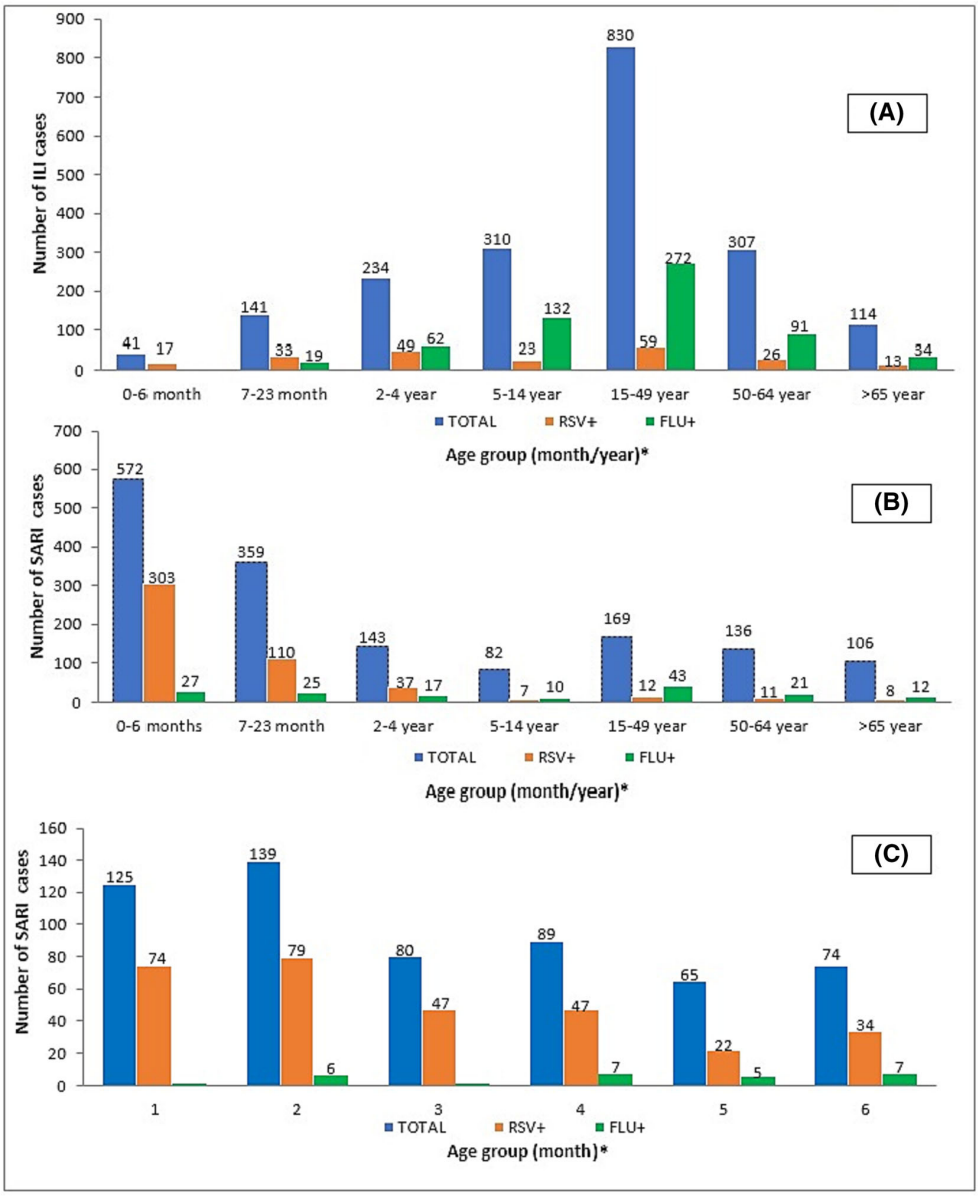
Positive cases for Influenza A/B (FLU) and HRSV detected in Morocco during 2012 ‐ 2017 seasons detailed by age groups and SARI/ILI syndrome. The *X*‐axis shows the different categories of patient age groups. The Y‐axis displays the total number of enrolled SARI/ILI cases and the number of positive cases for Influenza A/B or HRSV. *Samples with missing data from age have not been included in the figure.

We found a higher proportion of HRSV infection in infants aged between 0–6 months old (44%; 320/726) followed by children aged between 7–23 months (20%; 143/726) and those aged between 2–4 years (12%; 86/726). Further, Influenza virus infection was most detected among adults aged between 15 and 49 years (39%; 315/812), followed by children aged between 5 and 14 years (17%; 142/812) and adults aged between 50 and 64 years (14%; 112/812). Positive cases for Influenza A/B and HRSV detailed by age group and syndrome are shown in Figure 1 and Table 2, respectively.

### 3.3 HRSV seasonal distribution

**FIGURE 2:**
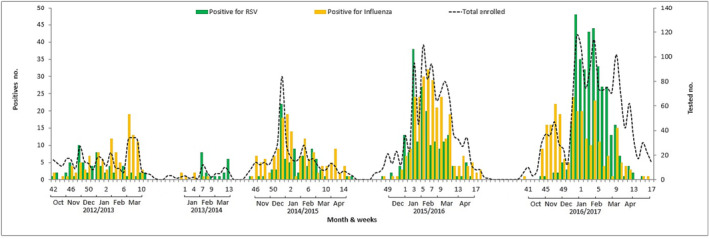
Circulation of HRSV and influenza virus between 2012 and 2017 in Morocco. The *X*‐axis shows the epidemiological weeks (EW) and months for each season. The primary *Y*‐axis displays the number of positive cases for each virus and the secondary *Y*‐axis shows the number of total enrolled samples.

For the period of study, the HRSV showed a clear periodic circulating pattern. Throughout 2012/13, 2014/15, 2015/16, and 2016/17 periods, the HRSV season started in the 47th epidemiologic week (November) of the year, peaked between the 52nd and 53rd week (December–January), and ended between the 10th and 13th week (March) of the next year. Season 2013/14 was an exception, owing to a fewer and time‐shorted sampling. Therefore, the circulation period of HRSV lasted for 16 to 21 weeks (Figure 2), and the detection rate for HRSV‐positive infection was highest during the fourth and the first quarter of each season (Table 1; *P* < 0.001).

### 3.4 Prevalen and distribution of HRSV subtypes

**FIGURE 3:**
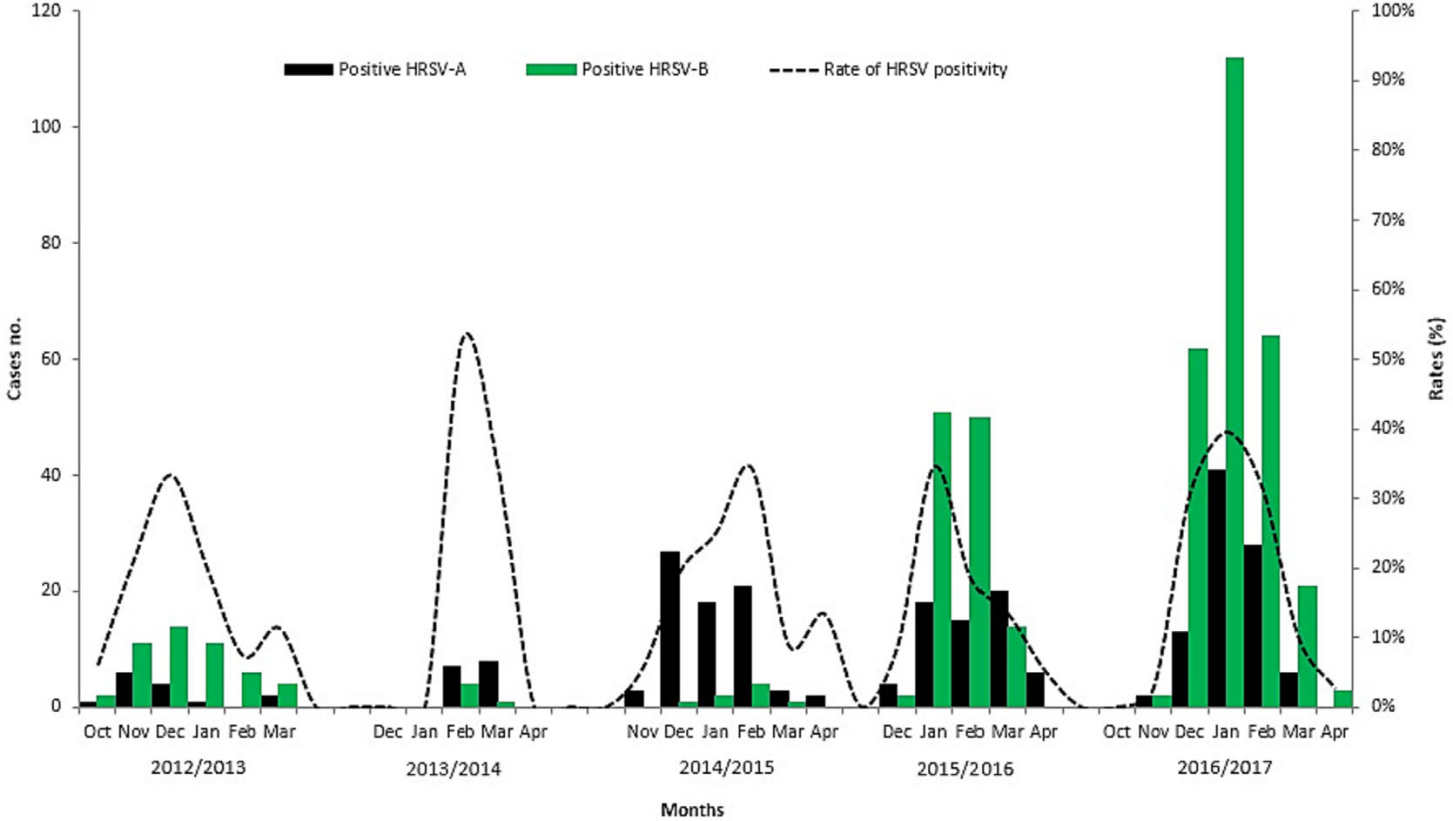
Circulation of HRSV‐A and HRSV‐B subtypes in Morocco between 2012 and 2017 seasons. The *X*‐axis shows the months for each epidemiological season. The primary *Y*‐axis displays the number of positive cases for each subtype and the secondary *Y*‐axis shows the evolution of HRSV positivity rate during the period of study.

From the total HRSV‐positive cases, 35% (257/726) were HRSV‐A subtype, the HRSV‐B subtype amounted to 61% (442/726) of the cases, and co‐infection was detected in 3.7% (27/726) of samples. Both subtypes co‐circulated during all study periods, with HRSV‐B predominating in three of the five seasons. The distribution of the HRSV strains is shown in Figure 3: 63 cases (14 HRSV‐A, 49 HRSV‐B) in 2012/13; 20 cases (15 HRSV‐A, five HRSV‐B) in 2013/14; 82 cases (74 HRSV‐A, eight HRSV‐B) in 2014/15; 182 cases (64 HRSV‐A, 118 HRSV‐B) in 2015/16; and 352 cases (90 HRSV‐A, 262 HRSV‐B) in 2016/17.

### 3.5 Phylogeny of HRSV and circulating genotypes

Full‐length HRSV gene sequences were obtained from 56 HRSV subtype‐A and 71 HRSV subtype‐B; eight samples failed in WGS owing to poor viral load. Successfully assembled sequences were deposited in the GISAID® database under assigned accession numbers EPI_ISL_15004431‐EPI_ISL_15004437, EPI_ISL_15120665‐EPI_ISL_15120779, and EPI_ISL_16905445‐EPI_ISL_16905450.

All HRSV‐A sequences from Morocco (2, 13, 9, 18, and 14 samples from 2012, 2014, 2015, 2016, and 2017, respectively) clustered in the novel ON1 genotype which has been described as having a common duplication of 72 nucleotides (nt) within the HVR2 region of the G protein gene, resulting in polypeptide elongated by 24 extra amino acids (GQEETLHSTTSEGYLSPSQVYTTS) spanning positions 284–307 compared to prototype strain A2.^9^

Sequence homology between Moroccan HRSV‐A sequences and the ON1 reference strain ON67‐1210A was ≥97.9% at the nucleotide level and ≥95.6% at the amino acid level, and the overall intra‐genotypic p‐distance was 1.4% for ON1 sequences.

The length of the G gene protein in all ON1 strains in this study was 322 amino acids (AA) with a TGA as a stop codon similar to the prototype ON1 strain ON67‐1210A [JN257693] from Canada. Ten of 16 previously described substitutions as universally identified in ON1 or NA1 genotypes when compared to reference strain HRSV‐A2 (S222P, P226L, E233K, T253K, I244R, L258H, S269T, P283S, P313S, and R321K) were conserved in all HRSV‐A from this study.^9^

**FIGURE 4:**
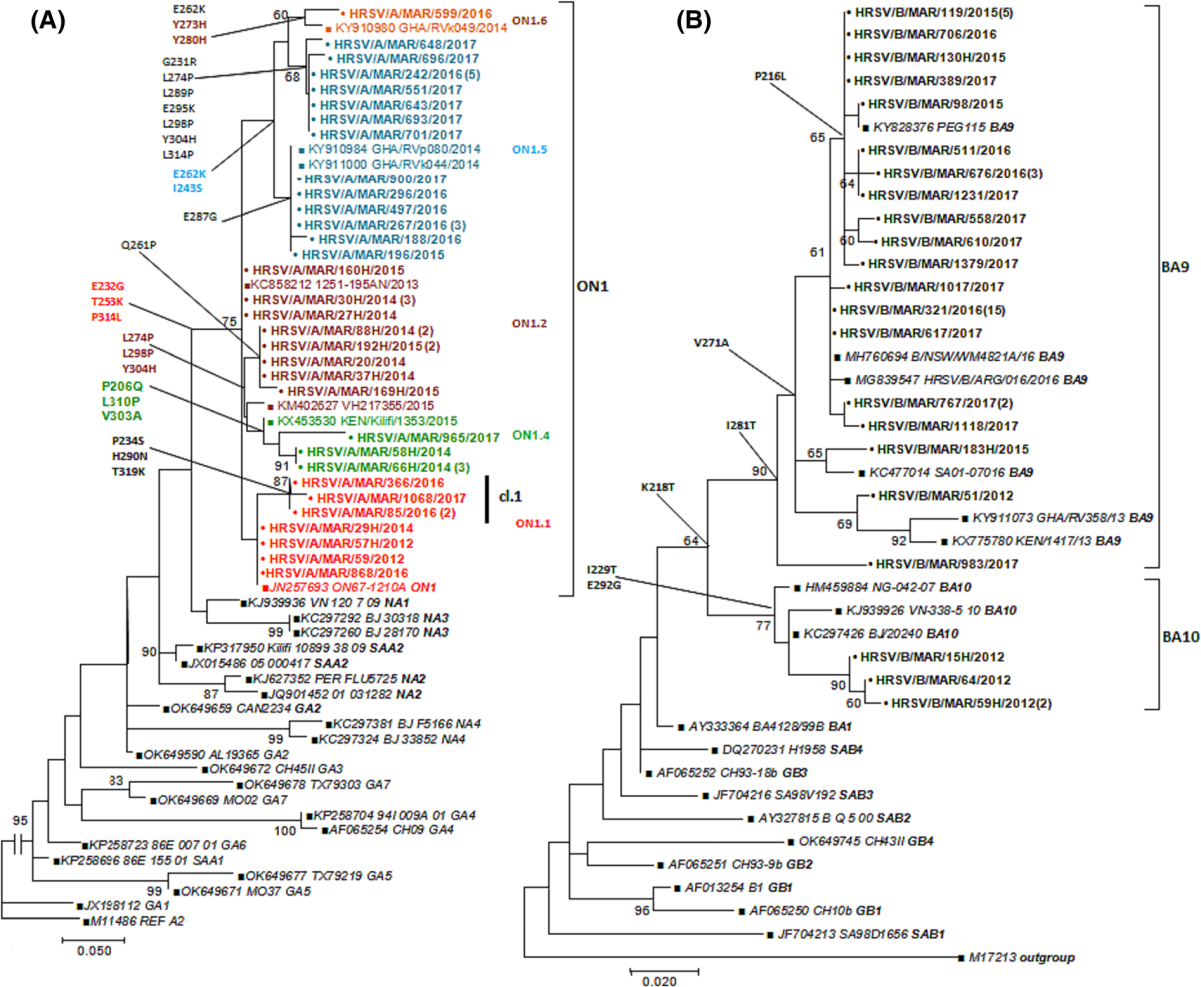
(A) Phylogenetic tree of the second hypervariable region of the G gene of HRSV‐A. (B) Phylogenetic tree of the second hypervariable region of the G gene of HRSV‐B. trees were constructed by the maximum likelihood method with 1000 bootstrap replicates using MEGA 7.0. Reference sequences of HRSV‐A or HRSV‐B genotypes from GenBank were included in the analysis and are indicated by their accession numbers. The reference genotypes are indicated in bold at the end of the isolate name. Moroccan HRSV viruses are in bold font. The genotypes are indicated by the brackets on the right side. Different font color indicates each phylogenetic subgroup within ON1 genotype. The number of identical sequences is indicated in brackets next to sequence name. Only bootstrap values above 60% are displayed at the nodes. Scale bar indicates nucleotide substitutions per site. Genotype/subgroup specific amino acid changes are reported along the tree branch. Cl = cluster.

Firstly, all Moroccan strains have identified three substitutions specific to genotype ON1 (E232G, T253K, and P314L). Within the ON1 genotype, five previously described phylogenetic subgroups (subgroup ON1.1: *n* = 8 strains; subgroup ON1.2: *n* = 12 strains; subgroup ON1.4: *n* = 5 strains; subgroup ON1.5: *n* = 19 strains and subgroup ON1.6: *n* = 1 strain), could be distinguished in the phylogenetic tree shown in Figure 4A on the basis of some amino acid substitutions in comparison with the ON1 prototype strain [JN257693].^9^, ^22^, ^23^

The first subgroup ON1.1 comprises isolates that are closely related to the primary Canadian strain with few mixed substitutions. Interestingly, we found within this subgroup four isolates clustering together with a bootstrap value of 87%, sharing P234S, T319K, and H290N substitutions which were unique to Moroccan strains; they were identified as Cluster1 (Figure 4A). The second subgroup ON1.2 aligned local strains with isolates from Italy and Spain [KC858212, KM402627] and was characterized by amino acid changes L274P, L298P, and Y304H. Strains of the ON1.5 subgroup shared two key amino acid substitutions, E262K and I243S, which were also seen in some strains from Ghana and Gambia [KY911000, KY910984]. While strains belonging to the ON1.4 subgroup were distinguished from other subgroups by a unique amino acid change P206Q. Further, the ON1.6 subgroup is characterized by unique amino acid changes at Y273H and Y280H/P in addition to E262K and I243S from the ON1.5 subgroup.^24^, ^25^

**FIGURE 5:**
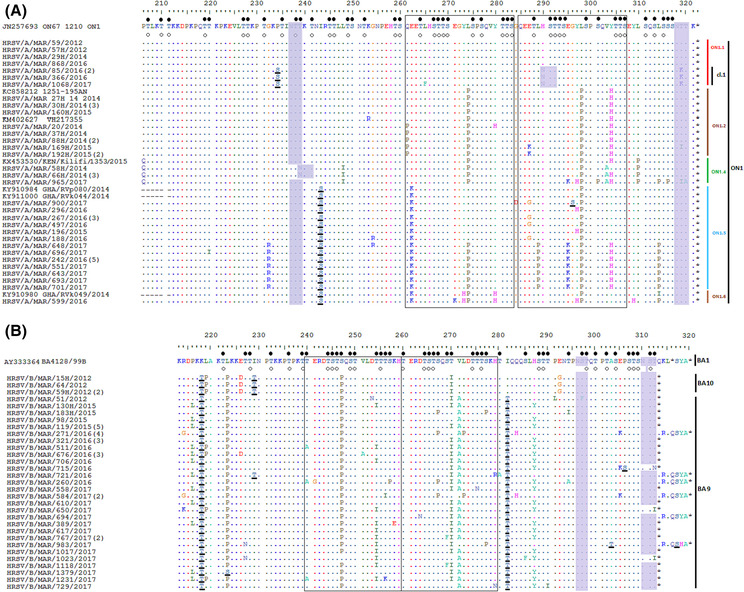
Alignment of deduced amino acid sequences of 2nd hypervariable region of Moroccan HRSV strains. (A) Alignment of HRSV‐A genotype ON1 sequences from Morocco and reference strains of known subgroups within the ON1 genotype. Alignments are shown and residues numbered relative to sequences of prototype ON1 strain [JN257693]. (B) Alignment of HRSV‐B viruses from Morocco in relation to prototype BA strain. Residues are numbered relative to the amino acid sequences of prototype BA strain [AY333364]. Number of duplicated sequences is indicated between parentheses (). Identical residues are indicated by dots, alignment gaps by dashes and stop codons by asterisks. Potential N‐glycosylation sites (N‐X‐T/S, where X is not a proline) are indicated by gray‐shaded rectangles. Potential O‐glycosylation sites of the prototype ON1 strain are indicated by black circles, while unfilled circles indicated the predicted O‐glycosylation sites common in all Morocco strains. Other predicted O‐glycosylation sites, not found in all the strains in this study are underlined. Identified subgroups within HRSV‐A ON1 or HRSV‐B strains are indicated in the side of alignment. Cl = cluster.

Analyzing the N‐glycosylation sites within the HVR‐2 of Morocco ON1 strains reveals that overall, they had two predicted N‐glycosylation sites at AA positions 237 and 318 and lost the N‐glycosylation site at AA position 251 due to a T253K/R substitution found in all ON1 strains. The change of amino acid threonine at position 239 into asparagine (T239N) caused a shift of the N‐glycosylation site from position 237 to position 239 in 4 samples. There was a unique H290N substitution that led to the gain of the N‐glycosylation site in four different strains assigned to cluster 1 in subgroup ON1.1 (Figure 5A).

The number of O‐glycosylation sites within the HVR‐2 of gene G in the investigated HRSV‐A sequences varies from 39 to 43 predicted sites and showed four additional potential O‐glycosylation sites when compared to the ON1 prototype strain (due to P234S, I243S, L274S, and G296S AA substitutions). The duplication region contained a maximum of 10 glycosylation sites, as observed in the ON67‐1210A strain in 53 ON1 isolates from Morocco, except for three different strains due to substitution at S299N in one sample and S301P in two samples.

Almost all of the HRSV‐B strains (1, 4, 30, and 32 samples from 2012, 2015, 2016, and 2017 respectively) belonged to the BA9 genotype except for four sequences from the year 2012 which clustered in the BA10 genotype (Figure 4B). Sequence homology between Morocco HRSV‐B sequences and the BA reference strain [AY333364] was 95.3%–96.8% at the nucleotide level and 91.5%–93.7% at the amino acid level. The intra‐genotypic p‐distances in BA9 was 1.6%, while the overall intra‐genotypic p‐distance was 0.1% for BA10 sequences, and the estimated divergence between BA9 and BA10 groups was 4.1%.

HRSV‐B sequences were aligned with the BA reference strain from Argentina [BA AY333364], which contain a 60 nt duplication in the HVR2 region of the G gene, resulting in 20 additional amino acids representing the ancestral evolutionary strain for both HRSV‐B BA9 and BA10 genotypes.^26^ The length of G protein was 313 AA for the BA10 genotype and either 313 AA or 320 AA for the remaining BA9 strains (Figure 5B).

Almost all Moroccan BA strains had K218T, L223P, and S247P key substitutions (except two strains). While BA9 strains have been characterized with I281T substitution, the BA10 genotype had L219P, E226D, I229T, and E292G particular substitutions. HRSV‐B strains had two N‐glycosylation sites that the NetNGlyc 1.0 server predicted at amino acid positions 296 and 310. However, three strains from 2016 and 2017 did not meet the typical amino acid pattern for the N‐glycosylation site at AA position 310 due to T312N and T312I substitutions. The potential O‐glycosylation sites of BA strains varied between 40 and 45 predicted sites, 27 of which were shared with the BA prototype strain. Also, they showed six additional predicted sites compared to the reference strain [BA AY333364] due to K218T, I229T, I281T, L223S, A303T, and P306S AA substitutions.

## 4 DISCUSSION

The respiratory syncytial virus has long been recognized as a leading cause of lower respiratory tract infections, especially in young children and older adults.^27^ Since there is neither an effective specific drug nor a vaccine for HRSV infection,^28^ long‐term monitoring and collection of data on HRSV circulation and variation patterns can help predict the overall level and scale of the epidemic to produce effective control and prevention strategies.^29^ This study investigated the prevalence, seasonality, and clinical characteristics of HRSV infection in patients with SARI or ILI. As well, we conducted phylogenetic and molecular analyses of different genotypes of HRSV strains detected in Morocco from 2012 to 2017.

HRSV surveillance in the country was built on the established Influenza surveillance system; therefore, HRSV and flu A/B virus were detected simultaneously in patients with a SARI or ILI infection. While influenza was mainly detected in adults presenting with mild symptoms, pediatric SARIs were mostly attributed to HRSV infection; our findings mirror those from other countries where HRSV remains the most prevalent virus in infants younger than 6 months.^30^, ^31^

The overall prevalence of HRSV in respiratory samples was 19.7%. Gaymard et al. reported a prevalence of HRSV ranging from 12 to 18% over four consecutive seasons,^30^ while reports that looked at a younger population found even higher percentages.^31^, ^32^ Thus, the need for HRSV screening to ensure proper management of hospital admissions and to prevent nosocomial infections.^32^

Most (61%) HRSV‐positive samples were from subgroup B, where HRSV‐A accounted for 35%. The percentage of codetection was 3.7%. A study from Senegal described a 3.4% coinfection rate with both subgroups,^33^ while another report from Brazil found 1.4% of codetections.^31^ Generally, dual infections occur rarely in HRSV‐positive respiratory samples.^34^

The data we gathered from this study consolidate what we found previously,^13^ that cough was most likely related to clinical symptoms of HRSV infection rather than fever (84%; *P* < 0.001). In fact, WHO reported that nearly half of the HRSV‐infected children do not show fever as the presenting symptom^35^; therefore, some researchers recommend the WHO‐ARI case definition as more appropriate and highly sensitive for HRSV surveillance.^36^ Recently, WHO advocated in their global respiratory syncytial virus surveillance pilot based on GISRS, the recruitment of cases meeting the “Extended SARI” case definition which was defined as hospitalized illness with cough and onset of symptoms within the last 10 days or an acute respiratory infection without fever as criterion.^11^, ^37^, ^38^ This case selection method leads to higher sensitivity for both influenza and HRSV viruses. In contrast, case definitions including fever (i.e. SARI) would result in an underestimate of HRSV infection, especially for young age groups.^37^, ^38^

Previous research established that both HRSV‐A and HRSV‐B subtypes circulate concurrently during each season, with the predominant subtype changing from year to year.^3^, ^9^, ^39^ Based on our limited data, there was a shift in the predominant subtype in the population every two seasons. Such periodicity of respiratory syncytial virus epidemics has been noted by other scholars.^40^, ^41^ One possible explanation for this displacement of the predominant strain is improved homologous herd immunity after an epidemic, which favors heterologous variants to circulate in a subsequent season and sustains annual outbreaks of HRSV disease.^3^, ^42^

The circulation of HRSV exhibits a clear periodic pattern. The epidemic starts in November, peaks in January to February of the following year, and last until April. Similar seasonality is reported in temperate regions, where HRSV epidemics generally begin in November or December each year, peak in January or February, and last for 4–5 months.^3^, ^32^, ^42^

ON1 was the sole HRSV‐A genotype detected during the study period, similar to what has been previously found in countries from the Middle East and North Africa (MENA) region and other continents where NA1 was rapidly replaced by ON1 genotype.^22^, ^23^, ^29^, ^31^, ^43^ The ON1 strain was first detected in Canada in 2010^9^ and has since widely spread and displaced the previously dominant HRSV‐A/NA1 genotype.^24^ The phylogenetic analysis of our local HRSV‐A isolates, including the ON1 prototype strain ON67‐1210A and various strains of known ON1 sub‐genotypes, revealed that Morocco strains could be divided into five subgroups (namely: subgroups ON1.1; ON1.2; ON1.4; ON1.5 and ON1.6) with characteristic AA substitutions as previously described.^9^, ^22^, ^23^, ^24^, ^25^

Most of HRSV‐A sequences had two predicted N‐glycosylation sites within the HVR2 (237/239 and 318), while four sequences owned a maximum of three sites (237, 290, and 318). These highly N‐glycosylated strains belonged to the 2016/2017 season, clustered together with a bootstrap value of 87%, and shared three additional substitutions (P234S, T319K, and H290N) compared to other ON1 strains. AA positions 234 and 290 were previously identified as being under positive selection and described as possibly escape mutants selected with specific monoclonal antibodies (mAbs).^9^, ^44^, ^45^ Also, position 290 was described as exhibiting a “flip‐flop” pattern.^9^, ^45^ P234S and T319K mutations already occurred in diverse countries, whereas amino acid substitution H290N was observed for the first time. Accumulation of AA substitutions inside the G gene may not only result in the gain and loss of N‐glycosylation or O‐glycosylation sites but also lead to changes in the effectiveness of some antiviral drugs. Indeed, we found a HRSV‐A sequence from 2017 carrying the (I189T) substitution in the heparin‐binding domain, previously reported in G‐Protein drug‐resistant mutants.^46^

HRSV‐B/BA genotypes emerged in the late 1990s, spreading globally to become the predominant genotypes worldwide.^26^ The prevalent HRSV‐B genotype during the study period was BA‐9 (*n* = 67; 94%), while the BA‐10 genotype was detected only in the 2012 season. Researchers from Lebanon, Pakistan, Saudi Arabia, France, and southern China found that BA‐9 and BA‐10 were the prevailling HRSV‐B genotypes during 2010 to 2017.^29^, ^30^, ^43^ Another report from Japan described the BA‐10 as a short live genotype that circulated only for several months during 2012 to 2015.^39^ By observing amino acid changes within the HVR‐2 of gene G, compared to the reference strain [BA AY333364], we deduced a gradual accumulation of substitutions over time; shifting from 10 to 15 substitutions over the period 2012 to 2017. Notably, the change (Q313/STOP) occurred in the majority of BA sequences leading to G protein shortening to 312 amino acids. Position 313 has been described as positively selected.^45^ Glycosylation site changes were also observed within HVR‐2 of HRSV‐B/BA strains. A loss of the N‐glycosylation site at position 310 due to T312N/I substitution in three sequences and a tyrosine substitution at positions (218,229 and 281) resulting in the gain of additional new O‐glycosylation sites in BA9/BA10, BA10, and BA9, respectively. These additional substitutions, along with the duplication, might have increased the fitness of this genotype relative to other HRSV‐B genotypes.^47^

Despite extensive knowledge about managing HRSV disease in infants and children, developing preventive strategies remains limited. Palivizumab was for a long time the sole effective monoclonal antibody against HRSV fusion protein agreed for intervention to prevent the disease infection and whose use intended to preterm infants and children with congenital heart disease at high risk for severe lower respiratory tract disease (LRTD).^2^, ^28^ On August 2023, the US Food and Drug Administration (FDA) approved (Abrysvo™), the first HRSV vaccine for use in pregnant women to prevent (LRTD) and severe (LRTD) caused by HRSV in infants, the same vaccine was previously approved in May for the prevention of (LRTD) caused by HRSV in individuals 60 years of age and older.^48^

Some limitations need to be considered when interpreting the data presented. First, recruiting cases by SARI/ILI criteria would underestimate the prevalence of HRSV disease, particularly in children. Therefore, we need a more sensitive and specific case definition. In addition, clinical information was extracted from reporting forms, which often contained inconsistencies and missing data, which may lead to bias in the analysis. Furthermore, due to the small sample size during our study, we could not exclude the possibility of missing detection of other circulating genotypes. Despite those limitations, we believe our findings will provide significant insight into circulation patterns and the molecular epidemiology of HRSV in the Moroccan community. This study highlights potential areas for further research.

## 5 CONCLUSIONS

In summary, the present study underlines the importance of HRSV as a Leading respiratory virus that can be a life‐threatening pathogen for infants, especially in developing countries. It describes the virus circulation as exhibiting a periodic pattern where Epidemics occur during fall months through early spring. Molecular studies identified the novel strain ON1 as the sole HRSV‐A genotype circulating in Morocco during 2012 to 2017, all HRSV‐B prevailing in the country during the same period belonged to BA genotypes. Therefore, it is necessary to continue monitoring HRSV surveillance to detect genotypes newly circulating in the country alongside new variants with relevant mutations that can lead to changes in virus characteristics and affect either antiviral drug effectiveness or vaccine development.

## AUTHOR CONTRIBUTIONS

H. Oumzil, A. Barakat, F. El Falaki, and A. Bimouhen: designed the study; S. Triki, T. Benamar, A. Barakat, and A. Rguig: provided data and materials; Z. Regragui, F. El falaki, H. Ihazmade, and S. Benkerroum: Data collection and processing; A. Bimouhen: Data analysis and Writing the paper; Y. Bakri and H. Oumzil: reviewing the manuscript.

## CONFLICT OF INTEREST STATEMENT

The authors declare no conflicts of interest.

### PEER REVIEW

The peer review history for this article is available at https://www.webofscience.com/api/gateway/wos/peer-review/10.1111/irv.13203.

## ETHICS STATEMENT

The protocol was approved by the Ministry of Health for the objective of conducting surveillance of respiratory diseases with epidemic potential, in which participants remain anonymous, and therefore did not require an assessment of the ethics committee or IRB approval. Verbal consent was obtained from all patients.

## Data Availability

The data that support the findings of this study are available from the corresponding author upon reasonable request.
